# Public Instruction in German

**Published:** 1917-08

**Authors:** 


**Public Instruction in German.**

Since our entry into the war, protests have arisen all over the country, against the teaching of German in schools, especially public schools. We hold no brief for the German language beyond its spelling. In an entirely unprejudiced spirit, or at least with no prejudice due to belligerency, we may express the opinion that the genders, the strong declensions and conjugations, the multiple declensions of adjectives and the construction are as atrocious as English spelling and we do not know of anything worse that can be said regarding a language. But the objection to teaching German is, first of all, a silly form of hostility that will logically extend itself to some of the best music and literature of the world. Secondly, while the German language itself is not what we would wish it to be, it is, as intimated, the key to literature of the highest grade and of large scope—rivaled only by Greek, Latin and English—as well as to technical writings of all kinds, including medical, which are, on the whole, unrivaled in any other language. ''If that be treason, make the most of it.'' Thirdly, as a common sense matter of protection against espionage, and as a general preparation for military and economic problems, the more Americans understand the German language, the better.

